# Tinea corporis intrafamilial infection in pets due to Microsporum canis

**DOI:** 10.1590/S1678-9946202466030

**Published:** 2024-05-13

**Authors:** Karla Yaeko Sierra-Maeda, Fernando Martínez-Hernández, Roberto Arenas, Leticia Boeta-Ángeles, Luary Carolina Martínez-Chavarría, Sonia Fabiola Rodríguez-Colín, Juan Xicohtencatl-Cortes, Rigoberto Hernández-Castro

**Affiliations:** 1 Hospital General ‘‘Dr. Manuel Gea González’’, Servicio de Dermatología, Tlalpan, Ciudad de México, Mexico; 2 Hospital General “Dr. Manuel Gea González”, Departamento de Ecología de Agentes Patógenos, Tlalpan, Ciudad de México, Mexico; 3 Hospital General ‘‘Dr. Manuel Gea González’’, Servicio de Micología, Tlalpan, Ciudad de México, Mexico; 4 Hospital Juárez de Mexico, Gustavo A. Madero, Ciudad de México, Mexico; 5 Universidad Nacional Autónoma de México, Facultad de Medicina Veterinaria y Zootecnia, Departamento Patología, Coyoacán, Ciudad de México, Mexico; 6 Hospital Infantil de México Dr. Federico Gómez, Laboratorio de Bacteriología Intestinal, Cuauhtémoc, Ciudad de México, Mexico

**Keywords:** Microsporum canis, Tinea corporis, Dermatophytes, b*-tubulin* gene, RAPD

## Abstract

*Microsporum canis*, one of the most widespread dermatophytes worldwide, is a zoonotic microorganism that transmits infection from reservoirs such as cats and dogs to humans. This microorganism is associated with *Tinea corporis* and other clinical manifestations; however, few studies have used genetic surveillance to determine and characterize the process of zoonotic transmission. In this study, we show a clear example of zoonotic transmission from a cat to an intrafamilial environment, where it caused *Tinea corporis* by infection with *M. canis.* Molecular characterization using the b*-tubulin* gene and Random Amplified Polymorphic DNA analysis made it possible to determine that the six isolates of *M. canis* obtained in this study belonged to the same genetic variant or clone responsible for reservoir-reservoir or reservoir-human transmission.

## INTRODUCTION

*Microsporum canis* is one of the most widespread dermatophytes worldwide, affecting 20–25% of the human population, and its incidence continues to rise with a high prevalence in urban areas, causing local epidemics in families, schools, and small communities^1,2^. *Microsporum canis* is a zoonotic organism that transmits infection from reservoirs such as cats or dogs to humans and is associated with clinical manifestations such as *Tinea corporis* due to its ability to invade the stratum corneum and keratinized tissues^2^.

In Mexico, *Tinea corporis* represents 15% of the superficial mycoses and is among the 10 most common causes of dermatological consultation^3^. In South America, the frequencies are very similar to those in Mexico; in Argentina, a six-year study (2002–2007) carried out in Buenos Aires reported a 15.19% frequency, while a systematic review carried out in Brazil in 2021 reported a relative frequency of 10%^4,5^. Similarly, in Europe, a prevalence of 18.46% was observed in a study carried out in Germany from 2007 to 2013, and in Japan, an epidemiological study reported a *Tinea corporis* rate of 8.3%^6,7^.

Cats and dogs are the most representative reservoir hosts of *M. canis* and are asymptomatic to the infection, which is a critical factor in the epidemiology and transmission to humans, making it an important zoonosis; however, some animals may be symptomatic, showing hair loss, blisters, papules, scabs, dandruff, scales and crusts, erythema, follicular plugging, hyperpigmentation, abnormal nail growth, and pruritus^2^. Transmission to humans happens via direct contact with an infected animal or person or by asexual forms (arthroconidia) in the environment, resulting in a papule that subsequently forms an annular lesion by radiated extension of filaments. *Microsporum* ringworm is characterized by small, multiple, centrifugally growing plaques with an active border^1^.

Conventional techniques for laboratory identification of *M. canis* are based on the detection of hyphae and arthroconidia by direct microscopic examination of clinical samples using 20% KOH solution, followed by mycological culture and analysis of morphological and microscopic features. However, the identification of *M. canis* is time-consuming and not definitive due to the very similar phenotypic characteristics among *Microsporum* species. The use of molecular tools allows for rapid, specific, and definitive identification. There are several molecular markers used for this purpose, such as the internal transcribed spacer (ITS), the b-*tubulin* gene, or specific markers such as the chitin synthase 1 (CHS1) gene^2,8^.

In this study, we present a clear example of zoonotic transmission in which a cat transmitted *Tinea corporis* to an intrafamilial environment by infection with *M. canis.* This was achieved by molecular characterization and Random Amplified Polymorphic DNA (RAPD) analysis of the b*-tubulin* gene, which allowed us to confirm the transmission of the same microorganism between the infected animals and humans.

The authors obtained all appropriate patient consent forms. In the forms, the patients consented to the use of their images and to the publication of other clinical information in the journal. The patients understood that their names and initials would not be published and that every effort would be made to conceal their identities, although anonymity cannot be guaranteed.

### Ethics approval

The authors declare that the procedures followed were in accordance with the ethical standards of the committee on human experimentation (institutional and national) of the Hospital General Dr. Manuel Gea Gonzalez, Mexico City, Mexico (February 27, 2022; IRB Nº 06-67-2021) and with the Declaration of Helsinki of 1975 as revised in 2000.

## CASE REPORT

**Figure 1:**
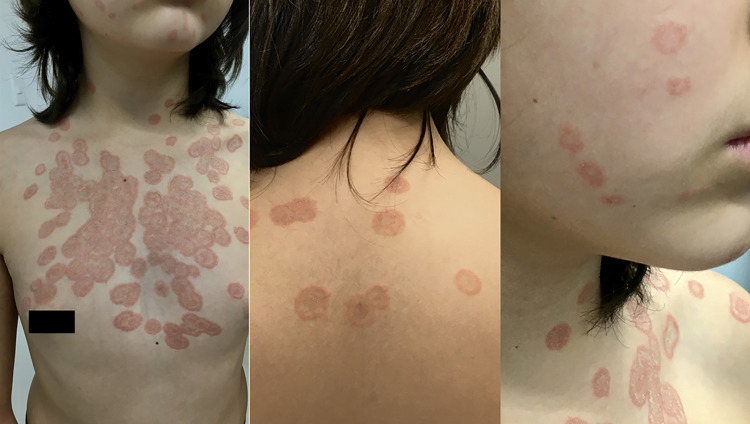
Erythematous and scaly round multifocal coalescent plaques on the chest, back, and face.

This study reports the case of an 11-year-old female with an 11-day history of multiple, annular, erythematous, scaly, and pruritic patches with an “active growing edge” and central clearing. These lesions affected the patient’s face, trunk, and back; they were of variable size (from 1 to 3 cm in diameter) and some were coalescent, forming larger lesions. The rest of the skin appendages showed no alterations (Figure 1).

Notably, the patient’s father and mother had similar lesions on the beard and left arm, respectively. They also had close contact with five animals: three dogs and two cats, of which a 3-month-old, recently adopted kitten was identified as the most likely source of infection because it was the first animal to present a characteristic fungal infection and other diseases, such as feline leukemia. The owners described this cat as having two erythematous-scaly lesions on its body, and within a few days the same lesions were observed on the rest of the animals. The animals received treatment based on ketoconazole shampoo every week, antiseptic solution, and povidone iodine on the lesions. Our patient had previously received treatment based on loratadine, diphenhydramine, and fluocinolone acetonide in unspecified doses due to the presumptive diagnosis of vascular or allergic disease, which led to a worsening of her clinical picture.

Due to the therapeutic failure, the patient consulted another physician, who suggested viral infection as the diagnosis and prescribed aciclovir 200 mg every 8 h. After three days of treatment, she presented with nausea, vomiting, and headache, for which the treatment was suspended without improvement. Finally, the patient consulted the mycology service and a presumptive clinical diagnosis of *Tinea corporis* was made. Treatment with terbinafine 250 mg every 24 h for one month was started for the whole family while awaiting the result of the mycological tests. Skin scrapings were taken from the clinical lesions of the patient and the five animals. Furthermore, some hair threads were taken from the animals for mycological analysis.

Direct microscopic examination was performed by preparing KOH 20% and examined microscopically for the presence of fungal elements. Fungal cultures were performed on Sabouraud dextrose agar (SDA) with 0.05% chloramphenicol and cycloheximide. The cultures were incubated at 25 °C for 15 days. A total of six strains were isolated from the 11-year-old patient and the five animals. On all media, fungal growth of whitish colonies with a chrome-yellow reverse, which were primarily identified as *Microsporum canis*, was observed. The isolates were identified as follows: 29 (the 11-year-old patient); 30 (dog #1); 31 (cat #1); 32 (cat #2); 33 (dog #2); and 34 (dog #3).

Molecular identification was performed using the partial sequence of the b-*tubulin* gene. Genomic DNA was isolated from pure cultures of six strains using a PureLink^®^ Genomic DNA kit (Invitrogen, Carlsbad CA, USA) in accordance with the manufacturer’s instructions. Polymerase Chain Reaction (PCR) was performed using a previously reported set of primers (TUBF 5’AACATGCGTGAGATTGTAAGT-3’ and TUBR 5’-TCTGGATGTTGTTGGGAATCC-3’), amplifying a 540 bp fragment^8^.

The PCR products were visualized on a 1.5% agarose gel stained with ethidium bromide; the amplicons were purified, and the nucleotide sequence was determined in both directions with Taq FS Dye Terminator Cycle Sequencing Fluorescence-Based Sequencing and analyzed in an Applied Biosystems 3730 xl DNA sequencing system.

Molecular identification of the isolates was performed by consensus sequence homology search in the GenBank database (Nucleotide BLAST). All six sequences showed 100% (452/452 bp) of genetic identity with b-*tubulin* of *Microsporum canis* (DQ449623). The sequences obtained were submitted to the GenBank database under accession numbers OL660657 (GEA-29), OL660658 (GEA-30), OL660659 (GEA-31), OL660660 (GEA-32), OL660661 (GEA-33), and OL660662 (GEA-34).

**Figure 2:**
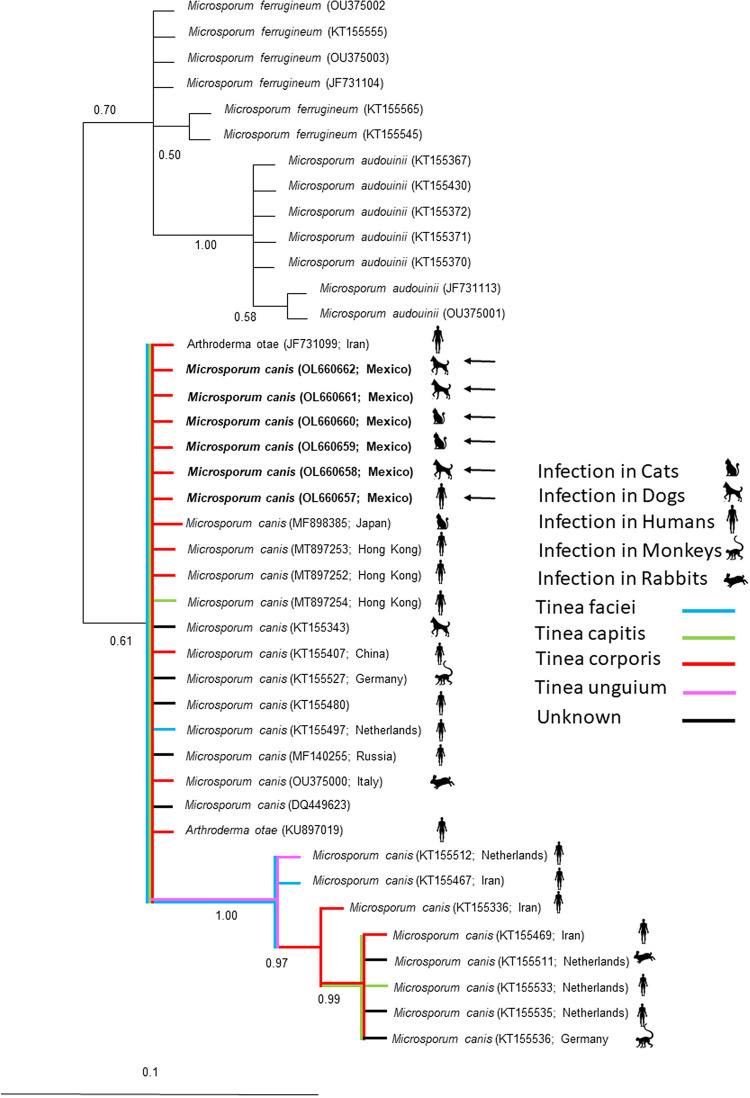
Bayesian phylogenetic tree using a partial sequence of the b*-tubulin* gene for *Microsporum canis*. The numbers of the nodes indicate the values of support or posterior probability. Samples obtained in this study are indicated in bold and by arrows.

Phylogenetic identification was performed by multiple alignments using MEGA X software^9^ with the sequences obtained in this study (OL660657-62) along with the sequences of *M. canis* from GenBank (JF731099, MF898385, MT897253-4, KT155343, KT155407, KT155527, KT155480, KT155497, MF140255, OU375000, DQ449623, KT155512, KT155467, KT155336, KT155536, KT155469, KT155511, KT155533, KT155535, and KU897019) and, as an outgroup, the sequences of *Microsporum ferrugineum* and *Microsporum audouinii*. Phylogenetic analysis was performed using a Bayesian model in Mr. Bayes software version 3.2.7^10^. The resulting consensus trees showed that the sequences obtained in this study were grouped in a specific clade, with *M. canis* having a posterior probability of 1.00 (Figure 2). Interestingly, the *M. canis* clade showed three genetically separated subclades, apparently related to the type of infection.

**Figure 3:**
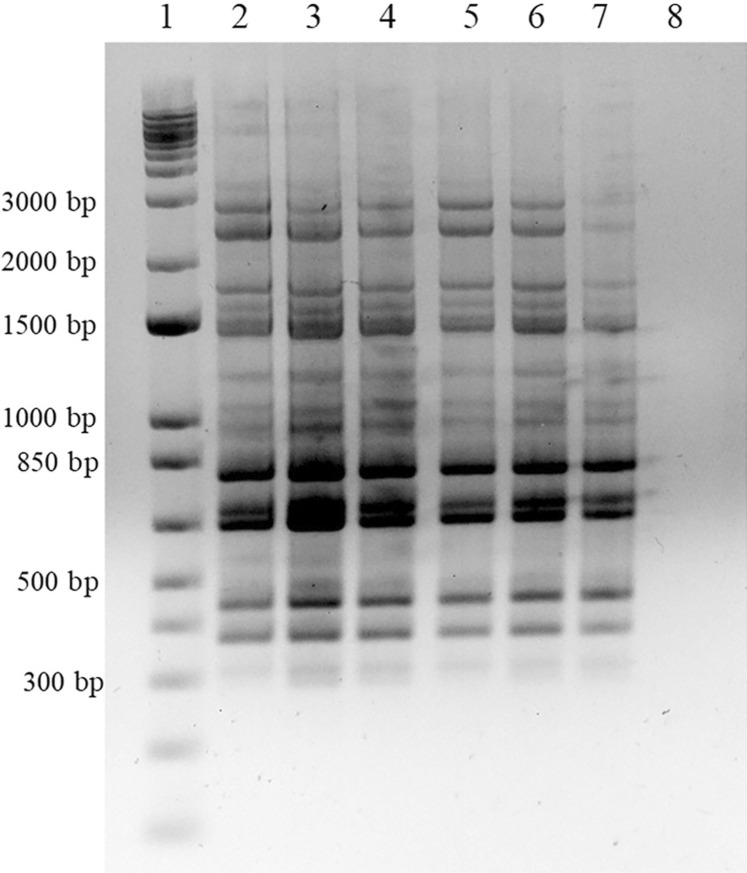
RAPD profiles were obtained using OPE-18 primer. All of them showed the same band pattern. Agarose gel electrophoresis of the amplified fragments. Lane 1: Molecular size marker 1 Kb Plus DNA ladder; Lane 2: *M. canis* GEA-29 OL660657; Lane 3: *M. canis* GEA-30 OL660658; Lane 4: *M. canis* GEA-31 OL660659; Lane 5: *M. canis* GEA-32 OL660660; Lane 6: *M. canis* GEA-33 OL660661; Lane 7: *M. canis* GEA-34 OL660662; Lane 8: Negative control.

In order to determine whether all isolates were the same variant of *M. canis* and thus confirm zoonotic transmission, a RAPD method was performed using the previously described decamer primers OPA-18 (5’-AGCTGACCGT-3’) and OPE-18 (5’-GGACTGCAGA-3’)^11^. This analysis showed that all isolates had the same genetic characteristics (Figure 3), evincing that the same fungal variant was transmitted between humans and companion animals.

## DISCUSSION

Approximately 40 species within seven genera (*Trichophyton, Epidermophyton, Nannizzia, Lophophyton, Paraphyton, Microsporum*, and *Arthroderma*) have been identified in infections caused by dermatophytes. These species have been classified as zoophilic, geophilic, and anthropophilic based on ecology, life cycle, and host preference^1^. *Microsporum canis* belongs to the group of zoophilic species, and human infections are usually caused by close contact with infected animals, such as cats and dogs, which have high infection frequencies depending on the geographical area^1,2^. Most of these infections are asymptomatic in dogs and cats and therefore represent a risk factor for humans unknowingly exposed to *M. canis*. When outbreaks occur, fomites and human-to-human or human-to-animal contact play an important role in dissemination.

Examples of *M. canis* transmission from dogs and cats to their owners have been reported in several countries, including Mexico^3^. In an epidemiological study conducted in Japan, different genotypes were identified in strains isolated from dogs and cats from pet shops and outdoor reservoirs, as well as in pet owners^12^. Similarly, in a military base in Israel, *M. canis* strains isolated from cats and soldiers shared identical genotypes, and in Spain, transmission of *M. canis* from adopted cats to their owners and pets was reported^13,14^.

In this study, we present a clear example of zoonotic intrafamilial transmission in Mexico City, where a cat carrying *M. canis* was adopted and subsequently caused *Tinea corporis* in three people and five animals. Mexico City has a population of 9.5 million and is located within the metropolitan area of the Valley of Mexico, which has a population of approximately 22.5 million. The distribution pattern of dermatophyte infections in this area shows a *Tinea corporis* incidence of 15%^3^.

Traditional mycological identification of *M. canis* is obtained mainly by examination of the lesion, microscopic observation of cultures grown in selective media, and phenotypic identification by experienced personnel. However, one of the major drawbacks is the time it takes to obtain the result (approximately 10–15 days), in addition to the fact that it may not be definitive because some isolates lack specific *M. canis* characteristics. Because of the increased infectivity in humans and animals, molecular diagnosis using tests such as PCR-sequencing, real-time PCR, RAPD, inter-single-sequence-repeat-PCR (ISSR-PCR), multilocus microsatellite typing (MLMT), and MALDI-TOF is necessary for correct classification and effective elimination, preventing further transmission^11,15,16^.

The b*-tubulin* gene has been reported to be an excellent marker for the identification of *M. canis* and other dermatophytes^11^. In this study, the analysis of this gene showed a high genetic variation, with the generation of three genetic subclades, one of them apparently of worldwide distribution, characterized mainly by the presence of clinical manifestations of *Tinea corporis*, while the other clades were found to be of Euro-Asian origin, with more diverse clinical manifestations (*Tinea capitis, Tinea corporis, Tinea unguium*, and *Tinea faciei*). Unfortunately, not much is known about the clinical manifestations and their genetic association; few reports suggest the existence of new highly transmissible pathogenic variants of *M. canis*, but more systematic studies are needed.

RAPD analysis is one of the most common, simple, rapid, and inexpensive molecular methods used in epidemiology^17^, which allowed us to determine that the six isolates of *M. canis* obtained in this study belonged to the same genetic variant or clone responsible for reservoir-reservoir or reservoir-human transmission.

Lastly, for the treatment of Tinea corporis, there are three main classes of antifungal medications used to treat dermatophytosis: allylamines (terbinafine, butenafine, naftifine), azoles (imidazoles and triazoles), and griseofulvin^18^. According to the literature, to treat systemic antifungal agents, experts prefer either terbinafine (250 mg once daily) or itraconazole (100 mg–200 mg/day) for two to four weeks of treatment^19,20^. We selected terbinafine for four weeks, which provided complete resolution of the infections.

## CONCLUSION

Our work introduces a clear example of zoonotic transmission from a cat to an intrafamilial environment, where it caused *Tinea corporis* due to infection with *M. canis*. Molecular characterization using the b-*tubulin* gene and RAPD analysis revealed that all *M. canis* isolates obtained in this study belonged to the same genetic variant or clone responsible for reservoir-human transmission.
